# Prognostic significance of GPR132 in papillary thyroid carcinoma: insights from integrated machine learning and its role in regulating TPC-1 cell growth

**DOI:** 10.1007/s12672-025-03833-0

**Published:** 2025-10-22

**Authors:** Jinghua Gao, Zihan Cai, Shoupeng Ding, Lanxin Ma, Jian Han, Yi-Yi Luo, Xueli Yang, Liqin Zhou, Wen Mei, Xiangfang Li, Lin Meng, Heng Luo

**Affiliations:** 1 Center for Precision Medicine, The People’s Hospital of Chuxiong Yi Autonomous Prefecture Chuxiong Hospital, Chuxiong, 675000 Yunnan China; 2 Department of Medical Laboratory, Siyang Hospital, Siyang, 237000 China; 3 Department of Laboratory Medicine, Gutian County Hospital, Gutian, 352200 China; 4 Pathology department, The People’s Hospital of Chuxiong Yi Autonomous Prefecture, Chuxiong, 675000 Yunnan China; 5 Scientific research management division, The People’s Hospital of Chuxiong Yi Autonomous Prefecture, Chuxiong, 675000 China; 6 thyroid surgery, The People’s Hospital of Chuxiong Yi Autonomous Prefecture, Chuxiong, 675000 Yunnan Province China

**Keywords:** Single-cell sequencing, Papillary thyroid carcinoma, GPR13, TPC-1 cell

## Abstract

**Object:**

This study utilizes machine learning and bioinformatics methods to analyze data identifying GPR132 as a reliable potential prognostic gene for papillary thyroid carcinoma (PTC).The experiments elucidated potential role of GPR132 in inhibiting tumor growth in PTC by regulating the cell cycle and apoptotic mechanisms. This research provides significant insights for future personalized therapeutic strategies aimed at targeting PTC.

**Methods:**

The study analyzed the GSE191288 RNA-seq dataset, which included six thyroid cancer tumor samples and one adjacent normal tissue sample, to identify genes associated with tumor-associated macrophages (TAMs). After conducting a thorough enrichment analysis, we used the CellChat tool to investigate the signaling pathways.Pseudotemporal analysis elucidated the differentiation status of TAMs, and weighted gene co-expression network analysis(WGCNA) identified M1-like TAM-related genes within the M1 macrophage module. Integration with the GEO database revealed that GPR132 is a key prognostic gene. The effects of GPR132 overexpression on the proliferation, migration, apoptosis, and cell cycle progression of thyroid papillary carcinoma (TPC-1) cells were evaluated through cell-based experiments.

**Results:**

Single-cell sequencing revealed 20 distinct cell clusters, categorized as epithelial, stromal, or immune cells, with a focus on TAMs.Enrichment analysis associated TAM-expressed genes with immune response regulation. Pseudotime analysis identified TAMs differentiation states, while WGCNA linked a low abundance of M1 macrophages to favorable PTC prognosis. Integration with the GEO database confirmed GPR132 as a key prognostic gene. Cellular experiments showed that GPR132 overexpression markedly inhibited TPC-1 cell proliferation and migration, likely through G1 phase cell cycle arrest and enhanced apoptosis. Flow cytometry confirmed elevated early and total apoptosis rates in GPR132-overexpressing cells.

**Conclusion:**

GPR132 was identified as a critical prognostic gene for PTC, with evidence suggesting its role in tumor suppression via cell cycle modulation and apoptosis induction.

## Introduction

 Recent cancer statistics indicate that thyroid cancer is the most prevalent form of endocrine malignancy in humans [1]. The rapid rise in thyroid cancer incidence is well-documented, with nearly a fourfold increase over the past 25 years in the United States, resulting in approximately 2,230 annual deaths [2].In South Korea, the incidence has surged approximately 15-fold [2]. In China, the incidence of thyroid cancer significantly increased from 2000 to 2016,with approximately 202,600 new cases reported in 2016 [3]. Among thyroid malignancies, papillary thyroid carcinoma (PTC) is the most common type, comprising approximately 80% of cases [4].Although PTC generally has a favorable prognosis and slow progression, 4.3% to 28% of patients remain at risk of tumor recurrence [5]. The risks of invasion, metastasis, and recurrence are often underestimated, potentially resulting in poor prognosis and mortality [6–9]. Therefore, accurate prognostic assessment is essential for treatment planning and long-term management of PTC patients. These assessments not only guide therapeutic strategy selection—including surgery, radiotherapy, pharmacotherapy, or combined modalities—but also influence long-term predictions of disease progression and quality of life.In the era of personalized medicine, precise prognostic evaluation for each patient has become increasingly crucial [4, 10, 11].

Currently, the G protein-coupled receptor (GPCR) family has garnered increasing research attention due to its aberrant expression patterns in various tumors. As a member of the GPCR family, GPR132 also exhibits dysregulated expression across multiple tumor types, and its potential as a therapeutic target is receiving growing recognition within the scientific community [12, 13].G protein-coupled receptor 132 (GPR132) acts as a vital macrophage sensor, significantly influencing macrophages and cancer progression [7]. Increasing interest has focused on GPR132 expression across various tumor types and its potential as a therapeutic target; however, its specific roles in different cancers remain controversial.Studies suggest that GPR132 expression interacts with M2 macrophages in breast cancer, affecting cancer cell migration and macrophage activation [14–16]. Additionally, GPR132 expression has been positively associated with metastasis and poor prognosis in breast cancer [16]. In melanoma, GPR132 expression has been associated with tumor invasiveness [17]. In hematological malignancies, GPR132 activation inhibits BCR-ABL-induced acute B-cell lymphoblastic leukemia and demonstrates anti-tumor effects in acute myeloid leukemia(AML) [18, 19]. Despite these findings on GPR132 in various tumors, its potential mechanisms in papillary thyroid carcinoma (PTC) remain poorly understood.Thus, further investigation is required to elucidate its role in cancer progression.

This study utilized bioinformatics analysis to obtain single-cell sequencing data from the GEO database and employed R software to assess tumor cellular heterogeneity Subsequently marker genes were integrated with immune cell RNA sequencing data to establish a prognostic model for papillary thyroid carcinoma (PTC). Experimental results revealed that GPR132 may inhibit tumor growth in PTC by regulating cell cycle and apoptosis mechanisms. This research provides a novel molecular marker for the pathogenesis of PTC and offers significant insights for future personalized PTC therapies.

## Materials and methods

### Acquisition and preprocessing of Single-Cell data

The scRNA-seq dataset (GSE191288) included six papillary thyroid cancer (PTC) tumor samples, two for each from the same patient, for a total of three tumor patients. The dataset contains information on common PTC-associated driver mutations, including BRAF V600E and RET/FARP1 fusion, tumor stages (T1–T3), and patient gender. Additionally, a single adjacent normal thyroid tissue sample from one of these patients was included for comparison.To ensure data quality, the Seurat package in R version 4.3.1 was used for preprocessing. Cells with fewer than 300 detected genes and rare genes observed in fewer than three cells were excluded. Outlier detection was further performed using Grubbs’ test with a significance threshold of *p* < 0.05, and outliers were removed before downstream analyses. After quality control, a total of 29,561 cells were retained for further analysis. The SCTransform method was applied for normalization, followed by principal component analysis (PCA) for dimensionality reduction. The Uniform Manifold Approximation and Projection (UMAP) technique was used with the clustering resolution parameter set to 0.4. Using specific epithelial and immune cell markers, 20 distinct cell populations were identified.

### Cell annotation

Cell annotation was performed by integrating information from the CellMarker and SingleR packages, combined with relevant literature, to correlate cells with reference datasets and generate comprehensive annotations.Seurat clustered the cell populations based on PCA scores, using the “RunPCA” function on the top 2000 highly variable genes. The “FindNeighbors” and “FindClusters” functions were employed with a resolution parameter of 0.4 to refine the clustering. In this study, the classification of cell populations was conducted through comprehensive single-cell clustering analysis, supplemented by preliminary annotation utilizing established biomarkers. The clustering resolution was set at 0.4 to facilitate the identification of major cell types and certain subpopulations. Given the constraints of the data and analytical methodologies, this research did not pursue a more granular hierarchical classification of cellular subpopulations, including tumor-associated macrophages (TAMs). Future investigations could enhance the clustering resolution and incorporate functional validation to thoroughly explore and confirm the characteristics and functions of distinct subpopulations.

### Enrichment analysis

Marker genes were filtered using the “FindAllMarkers” function in R, with a log fold change(logFC) ≥ 0.25 and a minimum percentage of expression (minPct) ≥ 0.25. The top 200 highly expressed genes from each subpopulation were selected for enrichment analysis. Biological functions of these genes were visualized using the “ClusterProfiler” package in R.

### Cell communication analysis

The CellChat package was employed to analyze intercellular communication networks.The patchwork package integrated expression matrices to identify potential ligand-receptor pairs and their associated communication probabilities.

### Pseudotime analysis of cell trajectories

This study utilized single-cell RNA sequencing data and employed the Monocle 3 package to perform pseudotime analysis on tumor-associated macrophages (TAMs), aiming to elucidate their dynamic developmental processes and potential differentiation trajectories within the tumor microenvironment. Initially, highly variable genes were identified through variance analysis to capture cellular heterogeneity. Subsequently, rigorous quality control and standardized preprocessing were conducted, including the removal of low-quality cells and batch effect correction. Following this, dimensionality reduction techniques (such as UMAP and DDRTree) were applied to reduce data dimensionality and construct cellular developmental trajectories. Based on cellular expression profiles, pseudotime ordering algorithms were used to sequence cell states and infer their dynamic transition processes. Furthermore, by integrating pseudotime information, we identified genes significantly associated with temporal progression, analyzed their expression trends, and thereby revealed key molecular and functional changes in TAMs during tumor progression. Finally, Gene Ontology (GO) and pathway enrichment analyses were performed on these pseudotime-related genes to explore potential biological mechanisms.

### WGCNA for screening Macrophage-Related genes in PTC

The CIBERSORTx algorithm was applied to estimate the relative abundance of M1 and M2 macrophages in PTC samples. The R package “WGCNA” was used to identify hub genes highly correlated with macrophage activity.

### Machine learning for feature gene selection

In this study, Five machine learning methods—Boruta, LASSO, SVM, XGBOOST, and Random Forest (RF)—were applied to select feature genes. A Venn diagram was used to identify overlapping genes among the methods.

To evaluate model robustness, 10-fold cross-validation was performed. Among the models, the XGBoost classifier demonstrated the best predictive performance, achieving an average accuracy of 92.4% and sensitivity of 90.7% across validation folds. These metrics highlight the strong diagnostic potential of the selected ferroptosis-related feature genes.

### Immune infiltration and immune checkpoint analysis

The tumor immune microenvironment in high-risk and low-risk patient cohorts was analyzed using R packages such as CIBERSORT, ssGSEA, and ESTIMATE.Additionally, the correlation between risk scores and immune checkpoint-related gene expression was explored.

### Thyroid cancer cell line and transfection

This study employed a lentiviral vector system to establish a GPR132 stable transfection cell model. The accuracy of the GPR132-pLVX-EF1α-IRES-Puro recombinant plasmid was verified through sequencing. The experimental design included one untreated control group and two transfection systems: the experimental group involving co-transfection of the recombinant plasmid with packaging plasmids, and the empty vector group involving co-transfection of the empty vector with packaging plasmids. The mixed plasmids were transfected into packaging cells using Lipofectamine 3000, and the viral supernatant was collected after 48 h, followed by filtration and aliquoting for subsequent use. TPC-1 thyroid carcinoma cells (purchased from Cyagen, with STR authentication, were passaged to the third generation and seeded at a density of 2 × 10^5 cells/well in DMEM medium supplemented with 10% fetal bovine serum. The cells were pre-cultured for 20 h at 37 °C, 5% CO₂, and 98% humidity. Prior to transfection, the medium was replaced with Opti-MEM for a 4-hour serum starvation treatment. The experimental and control groups were treated with viral suspension at an MOI of 10, and the medium was replaced with complete medium after 4 h. Following 72 h of infection, the cells were subjected to puromycin selection at 90 ng/mL for 10–14 days. Monoclonal colonies formed in the experimental group, and stable clones were obtained through limited dilution. The expression level of GPR132 mRNA was validated by qPCR.

### Detection of GPR-132 gene expression levels in transfected cell lines

RNA extraction from the TPC-1 cell line was performed using TRIzol reagent (Takara, Japan) according to the manufacturer’s instructions. RNA was reverse transcribed into cDNA using the Fast-King cDNA Synthesis Kit (Novoprotein, China). Quantitative PCR was conducted using the ABI 7500 Fast Real-Time PCR System and SYBR Green PCR Master Mix (Tiangen, China), with primer sequences listed in Table 1.Relative gene expression levels were calculated using the 2^(-ΔΔCt) method based on the Ct values of β-actin and GPR132.

### Cell proliferation assay

Cell proliferation was evaluated in 96-well plates seeded with 3,500 cells per well. Three experimental groups were defined: TPC-1(Control), Empty Vector (EV), and GPR-132 overexpression. Cell viability was determined at different time points by measuring absorbance at 450 nm using the CCK-8 assay kit (Biosharp, China) according to the manufacturer’s instructions.

### Wound healing assay

Cells were seeded in 6-well plates and cultured to form a confluent monolayer. A scratch was created using a 200 µl plastic pipette tip. The cells were washed three times with PBS to remove debris, and serum-free medium was added to each well. Wound images were captured at predefined time points using an Ix71 inverted microscope (Olympus Corporation) and analyzed using ImageJ software. All assays were performed in triplicate.

### Transwell invasion assay

Invasion assays were performed using 24-well Transwell plates (pore size 8 μm, Corning, USA). The Transwell membrane was coated with 200 µg/ml Corning Matrigel and incubated at 37 °C for 5 h to allow gelation. 1 × 10^5 glioma cells suspended in serum-free medium were added to the upper chamber, while the lower chamber contained medium supplemented with 10% fetal bovine serum. After incubation at 37 °C for 6 h, the non-migrated cells on the upper surface of the Transwell membrane were stained with crystal violet, photographed, and quantitatively analyzed using ImageJ software.

### Flow cytometry

Apoptosis in the TPC-1 (Control), Empty Vector (EV) and GPR-132 overexpression groups was assessed using the Annexin V-FITC/PI apoptosis detection kit (Sevibio, China) with a flow cytometer. Additionally, cell cycle profiles of the three groups were analyzed using a cell cycle detection kit (Sevibio, China).

### Statistical analysis

All statistical analyses in this study were performed using Prism 9 (GraphPad Software, USA) and R software (version 4.3.1). For the comparison of continuous variables between groups, appropriate statistical methods were selected based on the distribution characteristics of the data: if the data conformed to a normal distribution, independent sample t-tests or one-way ANOVA were used; if not, non-parametric Mann-Whitney U tests or Kruskal-Wallis H tests were applied. Chi-square tests were used for the comparison of categorical variables. All statistical tests were two-sided, with a significance level set at *P* < 0.05. All cytological experiments were repeated three times to ensure the accuracy and reproducibility of the results.

## Results

### Single-Cell analysis of PTC

**Fig. 1:**
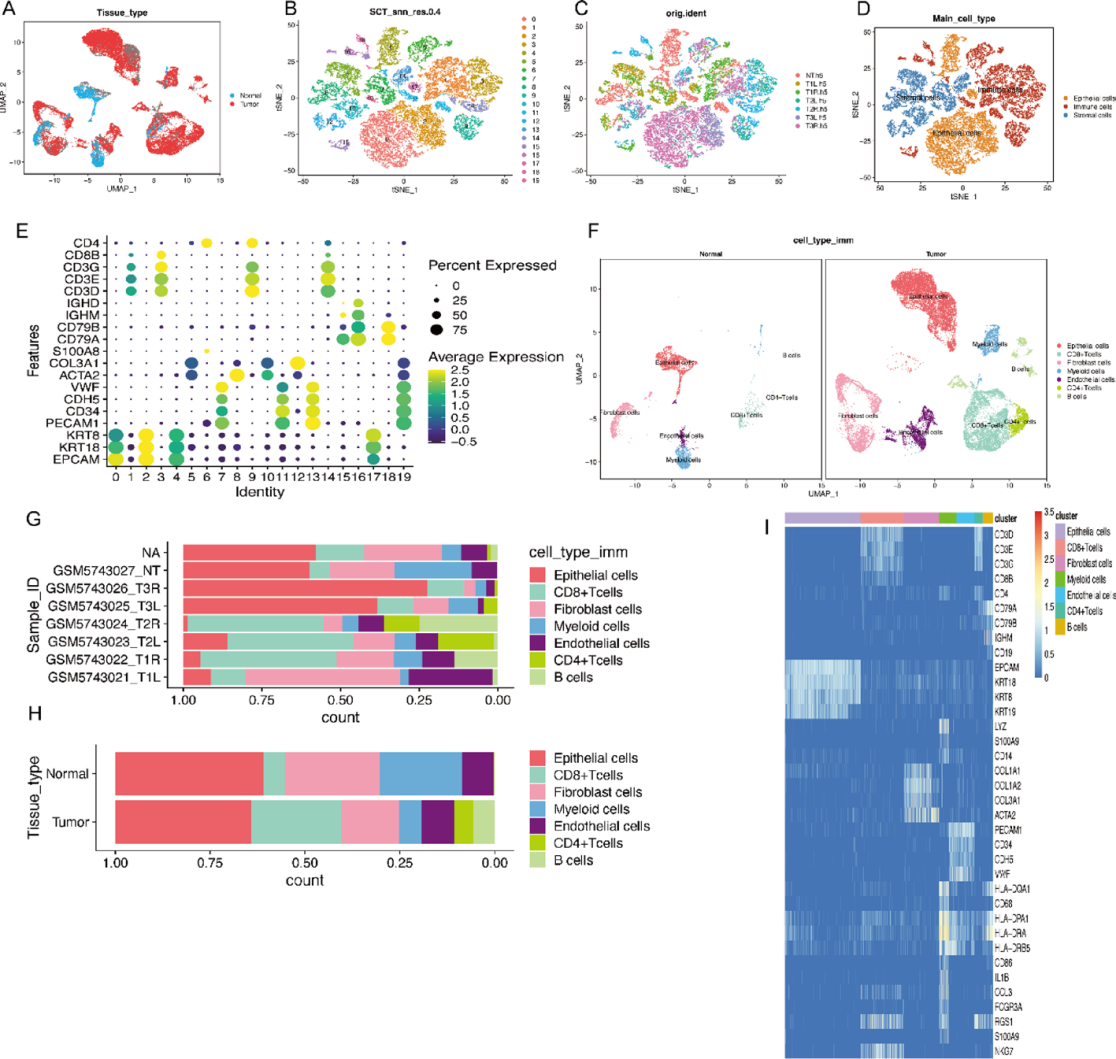
Single-cell RNA sequencing (scRNA-seq) analysis of thyroid cancer (THCA) and normal tissues.** A** UMAP visualization of tissue types. Red dots represent tumor samples, while blue dots represent normal tissue samples, illustrating the distinct clustering of tumor and normal cells.** B** t-SNE clustering based on SCT normalization. Cells are grouped into distinct clusters (*n* = 20), each labeled with a unique color.** C** t-SNE clustering by sample identity. Cells are colored according to their sample origin, including tumor and normal tissues, showing sample-specific clustering. **D** t-SNE clustering by major cell types. Cells are annotated into three main groups: epithelial cells (orange), immune cells (blue), and stromal cells (red), highlighting the major cell populations present.** E** Dot plot showing marker gene expression across clusters. The size of the dots represents the percentage of cells expressing the gene, while the color indicates the average expression level. Key immune, epithelial, and stromal cell markers are displayed, such as CD4, CD8A, EPCAM, and PECAM1.** F** UMAP plots showing immune cell distribution in normal (left) and tumor (right) tissues. Cell types include CD4 + T cells, CD8 + T cells, myeloid cells, fibroblasts, endothelial cells, and B cells. The tumor tissue exhibits a distinct immune cell composition compared to normal tissue. **G** Stacked bar plot showing cell type proportions by sample. Each bar represents a sample, and colors indicate different cell types. Tumor samples display a higher proportion of myeloid and fibroblast cells, while normal samples contain more B and T cells.** H** Stacked bar plot showing cell type proportions by tissue type. Tumor tissues exhibit a higher fraction of immune-suppressive cell types (e.g., myeloid cells), while normal tissues show a more balanced immune cell distribution.** I** Heatmap showing the expression of key genes across cell clusters. Clusters are labeled on the x-axis, while genes are listed on the y-axis. The color scale indicates expression levels, with higher expression in darker blue shades. Notable genes include CD3D, EPCAM, KRT18, S100A9, and NKG7, highlighting key differences between cell types

**Fig. 2:**
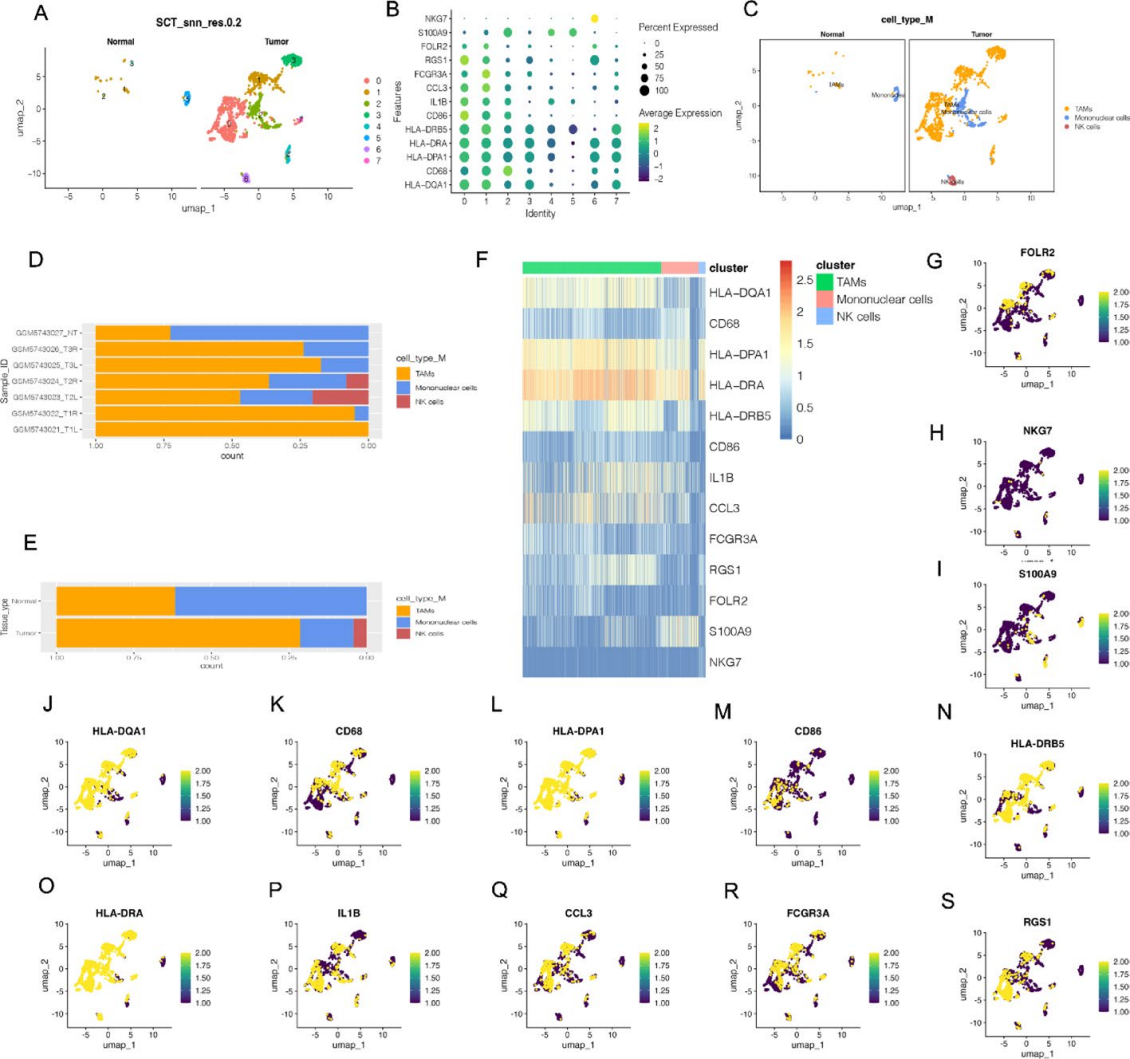
Single-cell RNA sequencing analysis reveals immune cell populations in tumor and normal tissues.** A** UMAP visualization of cell clusters in normal and tumor tissues. Cells from normal (left) and tumor (right) samples are shown. Each color represents a different cluster, highlighting the separation of cell populations between tissue types.** B** Dot plot showing marker gene expression across clusters. The x-axis represents different cell clusters, and the y-axis lists marker genes. Dot size indicates the percentage of cells expressing the gene, while color reflects the average expression level. Genes such as HLA-DQA1, CD68, and S100A9 are differentially expressed across clusters. **C** UMAP plot showing immune cell types in normal and tumor tissues. Immune cell populations are color-coded: TAMs (tumor-associated macrophages) in orange, mononuclear cells in blue, and NK cells in red. The tumor tissue shows an increased presence of TAMs. **D** Stacked bar plot of immune cell proportions across samples Each bar represents a sample, with colors indicating different immune cell types. Tumor samples contain a higher fraction of TAMs, while mononuclear cells are more abundant in normal tissues.** E** Stacked bar plot of immune cell proportions by tissue type The bar plot shows the overall proportion of immune cell types in tumor and normal tissues. Tumor tissues have an increased proportion of TAMs.** F** Heatmap showing the expression of marker genes across immune cell clusters The x-axis represents cell clusters, while the y-axis lists key marker genes. The color scale indicates expression levels, with higher expression in darker shades. Genes such as CD68, HLA-DPA1, and CCL3 are highly expressed in TAMs.** G**–**S** UMAP plots showing expression of selected marker genes Each plot displays the expression of a single gene across the UMAP embedding

This study analyzed seven samples, including six PTC tissue samples and one corresponding normal tissue control sample. Comparative analysis of the single-cell dataset between tumor and control groups was conducted (Fig. 1A). A substantial number of high-quality cells within the tissues were annotated using known marker genes. t-SNE and UMAP clustering identified 20 distinct subclusters, reflecting the heterogeneity of the tumor microenvironment (Figs. 1B and 2A). The single-cell data across different samples were visualized (Fig. 1C), and these cell clusters were categorized into three main groups: epithelial cells, immune cells, and stromal cells (Fig. 1D).

According to the literature, immune cells prominently express CD3E, CD4, CD79A, and CD3G, epithelial cells primarily express EPCAM and KRT18,and stromal cells predominantly express PECAM1 (Fig. 1E). These genes are fundamentally implicated in THCA pathogenesis and immune modulation. Based on marker gene expression, cells were further classified into types such as CD4 + T cells, CD8 + T cells, myeloid cells, fibroblasts, and endothelial cells (Fig. 1F). Additionally, the proportions of different cell populations across patients were visualized (Figs. 1G-I), revealing significant heterogeneity among cell types across different tumor samples.

To investigate the role of myeloid cells in THCA, we further clustered these cells using UMAP two-dimensional visualization techniques, identifying tumor-associated macrophages (TAMs), mononuclear cells, and natural killer (NK) cells as subgroups (Figs. 2A-C). The distribution of these subgroups across samples demonstrated that monocytes were predominantly found in normal tissues, while TAMs were significantly enriched in tumor tissues (Figs. 2D-E).

Further gene expression analysis revealed that TAMs exhibited high expression of key immune-suppressive and tumor-promoting markers such as CD68, FOLR2, and S100A9, supporting their role in tumor immune evasion and progression (Fig. 2F, G and I). In contrast, NK cells, which play a crucial role in anti-tumor immunity, were significantly reduced in THCA tissues, as indicated by the downregulation of NKG7 (Fig. 2H). Pathway enrichment analysis suggested that TAMs were associated with cytokine-cytokine receptor interactions, antigen presentation, and inflammatory response pathways, further emphasizing their immunosuppressive role in the THCA microenvironment.

Additionally, analysis of antigen-presenting gene expression (HLA-DQA1, HLA-DPA1, HLA-DRA, HLA-DRB5) in TAMs suggested a potential role in modulating adaptive immunity (Figs. 2J-O). Furthermore, pro-inflammatory cytokines such as IL1B and chemokines such as CCL3 were upregulated in TAMs, indicating their involvement in inflammatory signaling and immune cell recruitment (Figs. 2P-Q). The high expression of FCGR3A and RGS1 further supports the functional plasticity of these immune cells in shaping the tumor microenvironment (Figs. 2R-S).

These findings highlight the significant immune heterogeneity within THCA tumors, characterized by TAMs enrichment, NK cell depletion, and altered antigen-presenting gene expression. Such shifts in the immune landscape may contribute to tumor progression and immune evasion, providing potential targets for future therapeutic strategies.

### Gene enrichment analysis

**Fig. 3:**
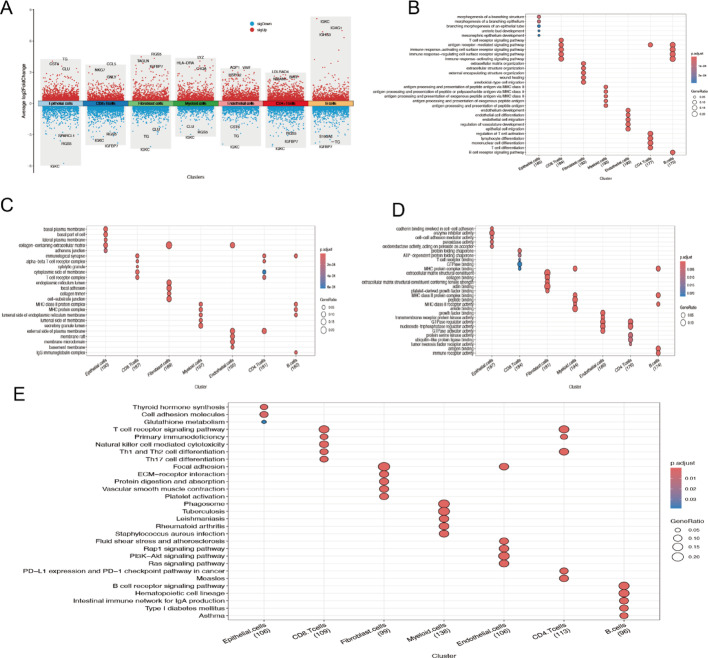
Cell Enrichment Analysis.** A** Differential analysis of cell populations.** B**-**D** Gene Ontology (GO) enrichment analysis.** E** Kyoto Encyclopedia of Genes and Genomes (KEGG) enrichment analysis

Gene Ontology (GO) and Kyoto Encyclopedia of Genes and Genomes (KEGG) enrichment analyses were conducted on highly expressed genes within TAMs subpopulations. Highly expressed genes were defined as those with an average log2 fold change (log2FC) > 0.25, an adjusted p-value < 0.05, and a detection frequency of at least 25% within the respective TAMs subpopulation. These genes were primarily associated with T cell activation, immune response regulation, and MHC-I/II pathways. Relevant genes were predominantly expressed in ribosomes and mitochondria and were associated with molecular functions like structural assembly, enzyme activity regulation, and death receptor activation. This suggests that TAMs exhibit heightened metabolic activity and immune surveillance capabilities. KEGG analysis further indicated associations between TAMs and immune-related conditions, including cell adhesion molecules, graft-versus-host disease, antigen processing and presentation, and autoimmune thyroid disease. These findings provide valuable insights into TAMs roles in immune-related diseases (Figs. 3A-E).

### Analysis of Cell-Cell interactions associated with TAMs in scRNA-seq

**Fig. 4:**
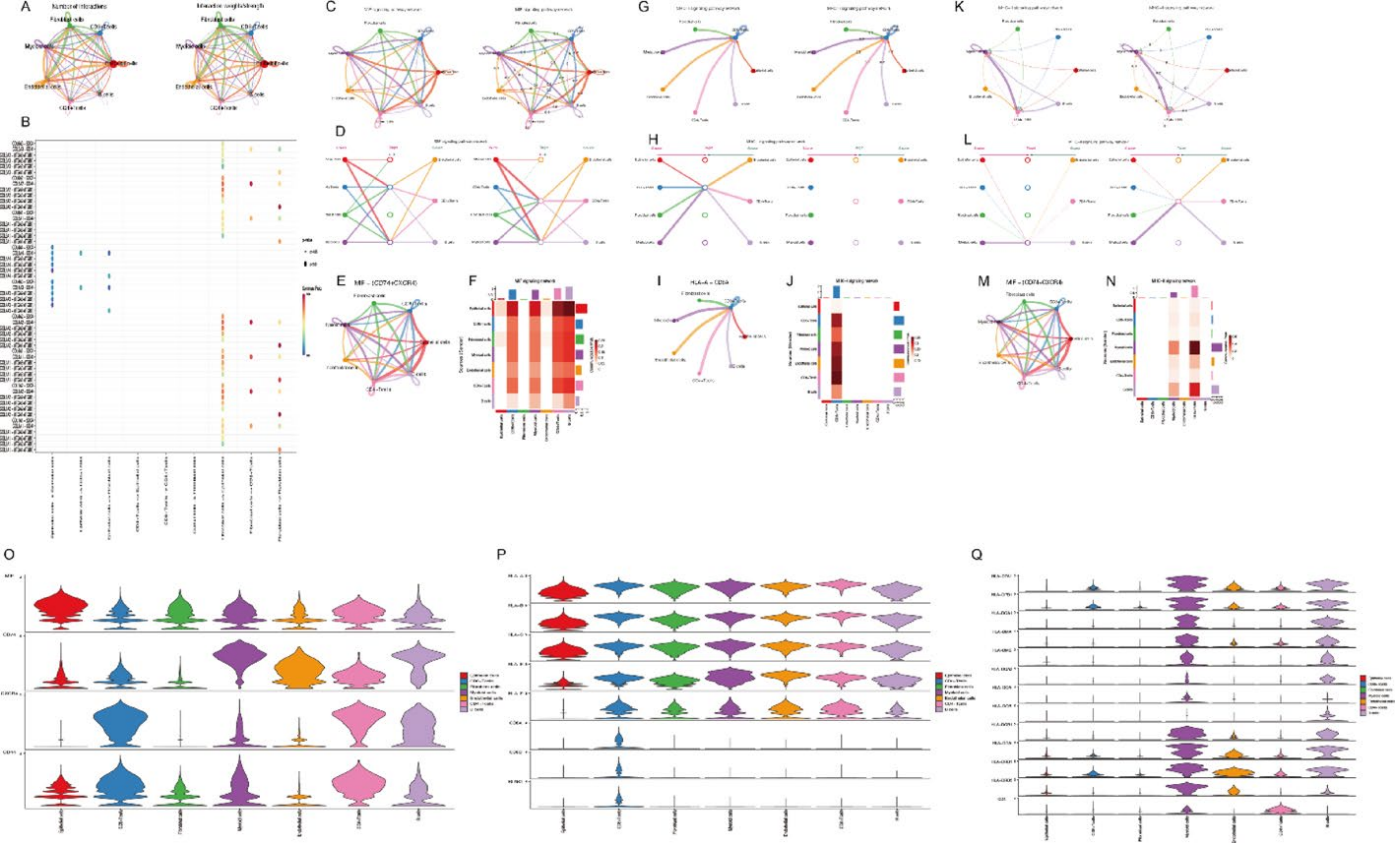
Cell-Cell Communication Analysis.** A** Intercellular interactions.** B** Receptor-ligand interactions across cell populations.** C** Interacting cells within the MIF signaling pathway. **D** MIF signaling network diagram. **E** Cell-cell communication network emphasizing MIF (CD74 + CXCR4).** F** Heatmap of MIF signaling across cell populations. **G**-**J** MHC-I signaling pathway analysis. **K**-**N** MHC-II signaling pathway analysis. **O**-**Q** Receptor/ligand gene expression levels

Cell surface receptor-ligand interactions provide insights into mechanisms of intercellular communication. By analyzing gene expression matrices, we inferred protein expression and delineated the cell interaction network. Using the R package “CellChat,” we explored specific signaling pathways and receptor-ligand interactions. Our analysis revealed that TNFRSF1A and MIF (CD74 + CXCR4) are key components in the TNF and MIF signaling pathways (Figs. 4A-Q).

### Analysis of TAMs cell maturation trajectories

**Fig. 5:**
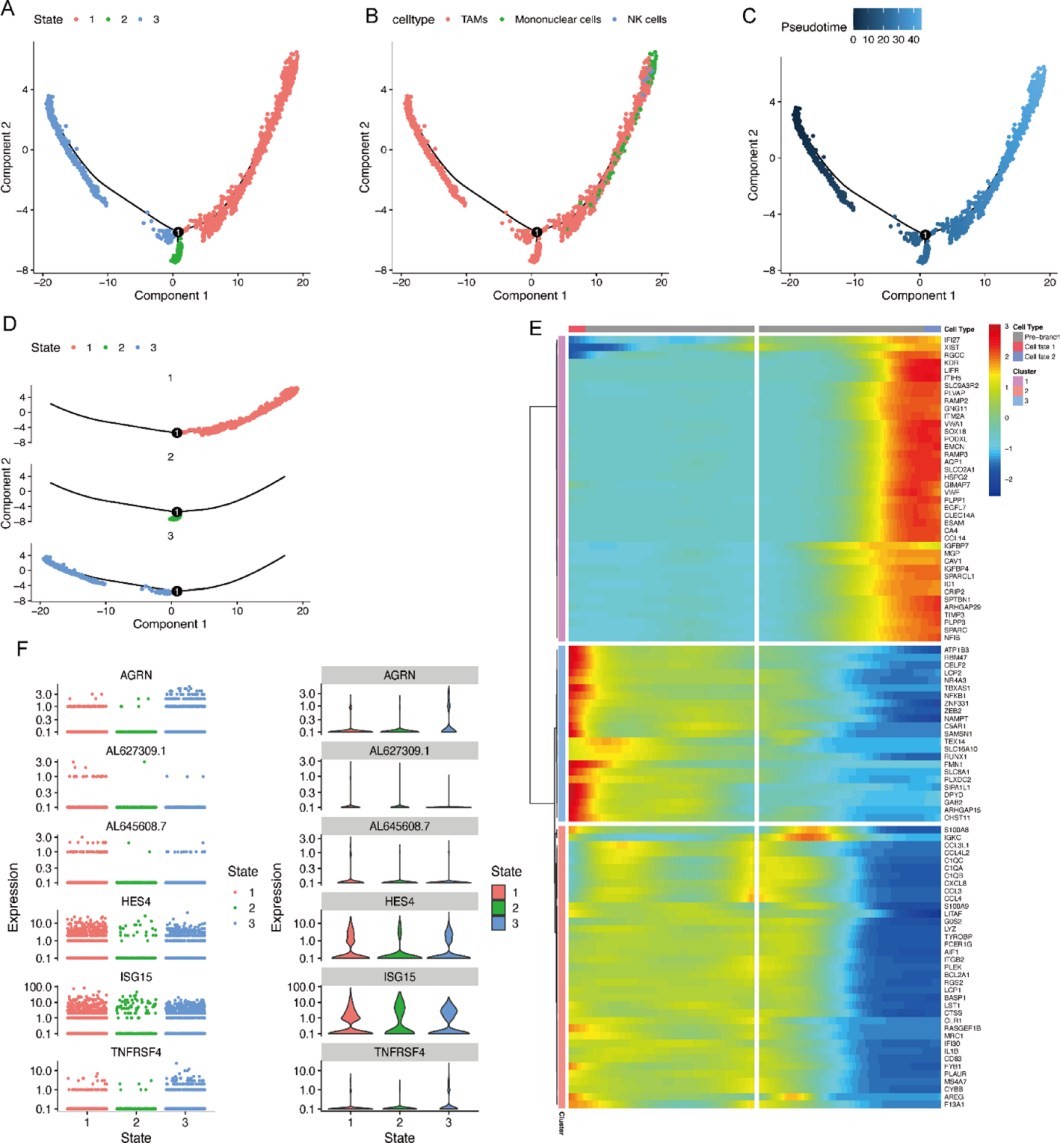
T Cell Trajectory Analysis. **A** TAM state distribution along the cellular trajectory. **B** TAM subtype distribution along the trajectory. **C** Temporal progression of TAM development. **D** TAM trajectory across different states. **E** Heatmap of differentially expressed genes. **F** Top 6 gene expression variations over pseudotime

Utilizing Monocle3 (v1.4.0) for pseudotime analysis, we successfully constructed a dynamic trajectory model of cellular development. The results demonstrate that the cellular subpopulations in the study samples exhibit a distinct three-stage differentiation pattern, corresponding to three major cell clusters (Figs. 5A-B). The temporal axis of cellular differentiation (Figs. 5C-D), as revealed by the color gradient representing developmental sequence, indicates that darker regions correspond to early developmental stages, while the gradual transition to lighter regions signifies progression toward terminal differentiation states. Monocle3 computed the overall differentiation sequence, with notable observation that TAMs persist throughout the entire developmental axis, suggesting their potential dynamic plasticity within the tumor microenvironment. The heatmap visualization illustrates the dynamic expression patterns of these genes along the pseudotime axis (Fig. 5E), with six genes demonstrating the most significant temporal expression variations being specifically annotated (Fig. 5F).

### Identification of Macrophage-Related genes in THCA via WGCNA

**Fig. 6:**
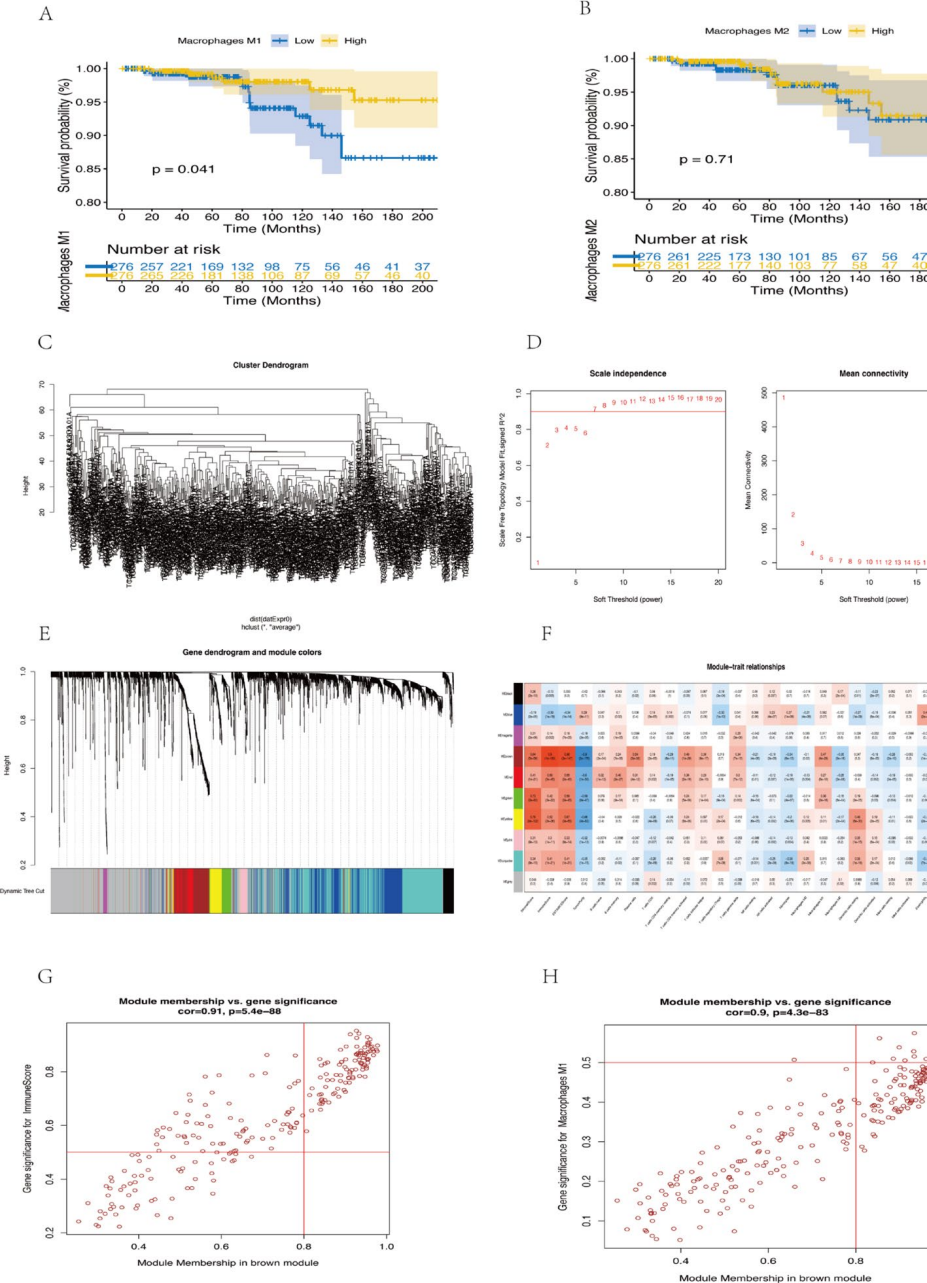
WGCNA Analysis.** A** Kaplan-Meier survival analysis for M1 macrophages.** B** Kaplan-Meier survival analysis for M2 macrophages.** C** Outlier analysis.** D** Optimal soft threshold determination for WGCNA.** E**-**F** Module correlation analysis. **G**-**H** Correlation analysis of the brown module

To investigate the relationship between macrophages and prognosis in THCA, we employed the CIBERSORTx algorithm to quantify M1 and M2 macrophage proportions in TCGA-THCA samples. THCA patients were categorized into high and low M1 macrophage content groups and high and low M2 macrophage content groups.Kaplan-Meier analysis indicated no statistically significant differences in overall survival between high and low M2 macrophage cohorts (Fig. 6B). However, patients with lower M1 macrophage content exhibited longer survival times (Fig. 6A), highlighting the role of M1 macrophages in THCA prognosis.Building on these findings, we performed Weighted Gene Co-expression Network Analysis (WGCNA) to identify genes associated with M1 macrophages. No outliers were detected in the TCGA-THCA dataset (Fig. 6C), and a soft threshold power of 7 was chosen (Fig. 6D), resulting in 10 modules (Figs. 6E-F). Correlation analysis revealed that the brown module was most significantly associated with high M1 macrophage content.A total of 323 genes from the brown module were selected for downstream analysis(Figs. 6G-H).

### Machine Learning-Based selection of prognostic genes

**Fig. 7:**
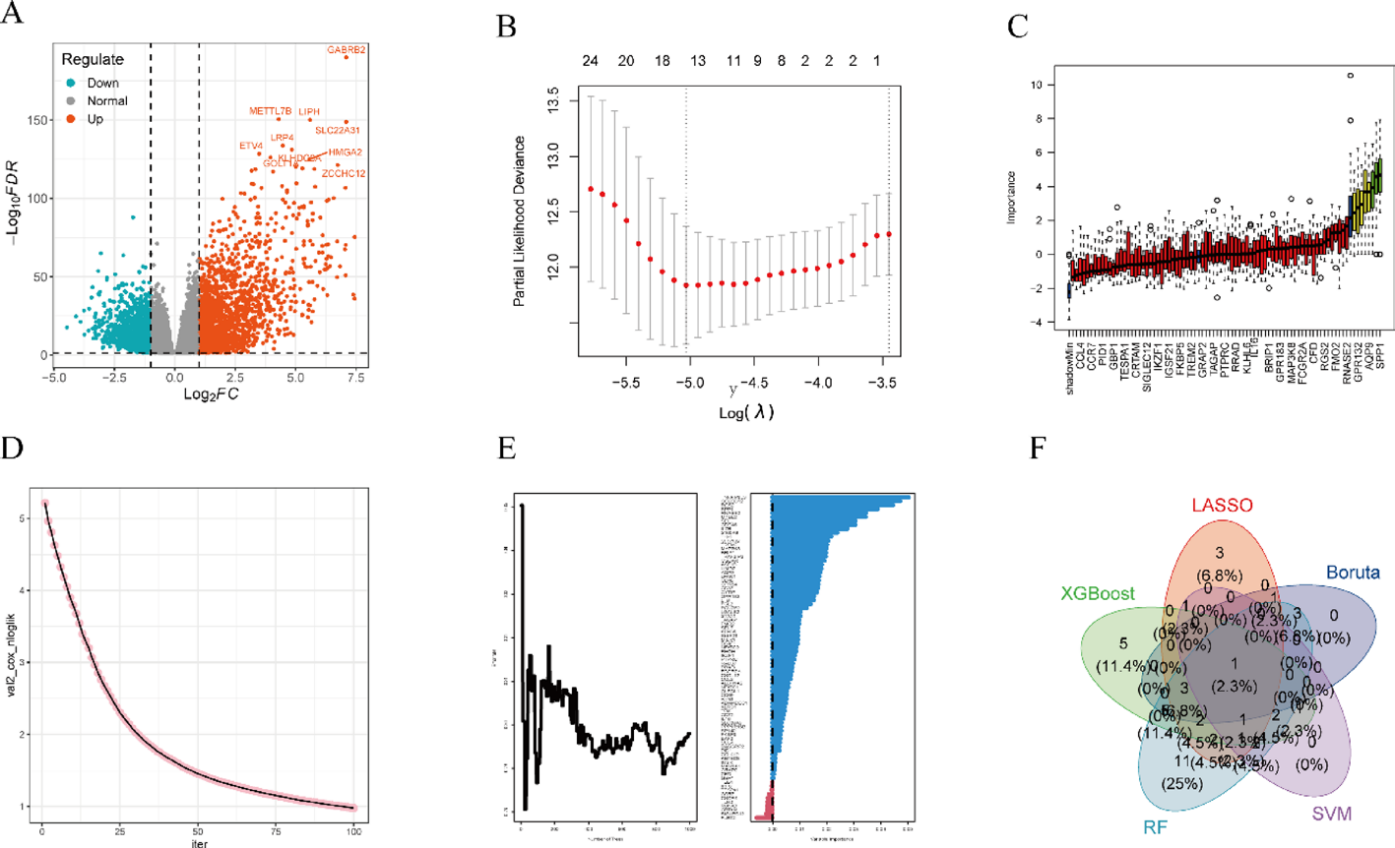
Machine Learning Selection of Prognostic Genes.** A** Volcano plot of gene selection.** B**-**E** Prognostic gene selection via machine learning. **F** Venn diagram showing intersection of selected genes

To identify prognostic genes, we applied a multi-step filtering process combining WGCNA and machine learning algorithms. First, we used WGCNA to select genes highly correlated with M1 macrophages, which were then cross-referenced with validated TAMs marker genes from single-cell datasets to identify potential biomarkers. This step identified 82 genes significantly associated with prognosis (Fig. 7A).

Next, we employed five machine learning algorithms (Boruta, LASSO regression, SVM, XGBOOST, and RF) (Fig. 7B and E) to perform feature selection. To ensure the robustness of the screening process, we used a Venn diagram to identify the common genes selected by all five algorithms. Among the intersecting genes, GPR132 emerged as a key prognostic gene due to its consistent identification across multiple models and superior predictive performance (Fig. 7F).

**Fig. 8:**
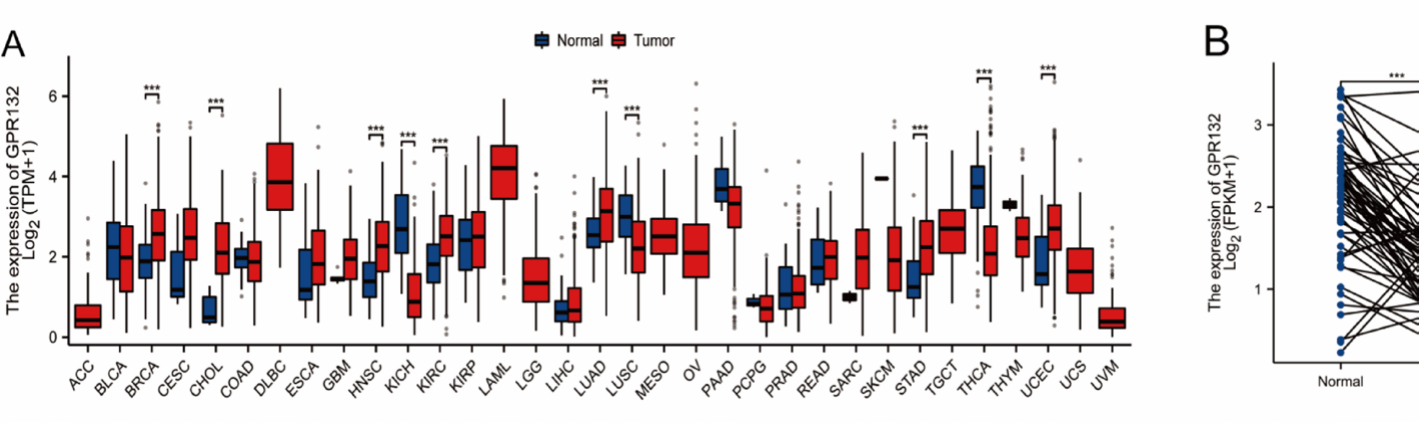
Expression of Prognostic Genes in Cellular Subpopulations.** A** Pan-cancer analysis of prognostic genes.** B** Prognostic gene expression levels

Pan-cancer analysis revealed that GPR132 exhibited significantly reduced expression in multiple tumor types, including thyroid carcinoma (THCA), kidney chromophobe carcinoma (KICH), and lung squamous cell carcinoma (LUSC) (Figs. 8A-B).These findings indicate that GPR132 exhibits strong prognostic performance and was thus selected as a representative prognostic biomarker.

### GPR132 enrichment analysis

The findings of this study have significant biological implications. First, this gene is highly enriched in multiple immune-related pathways, suggesting its crucial role in the recruitment, activation, and functional regulation of immune cells. Notably, its significant enrichment in the NF-κB and JAK-STAT signaling pathways indicates its involvement in the regulation of inflammatory factors and its potential role in chronic inflammation-related diseases, such as autoimmune and infectious diseases.

**Fig. 9:**
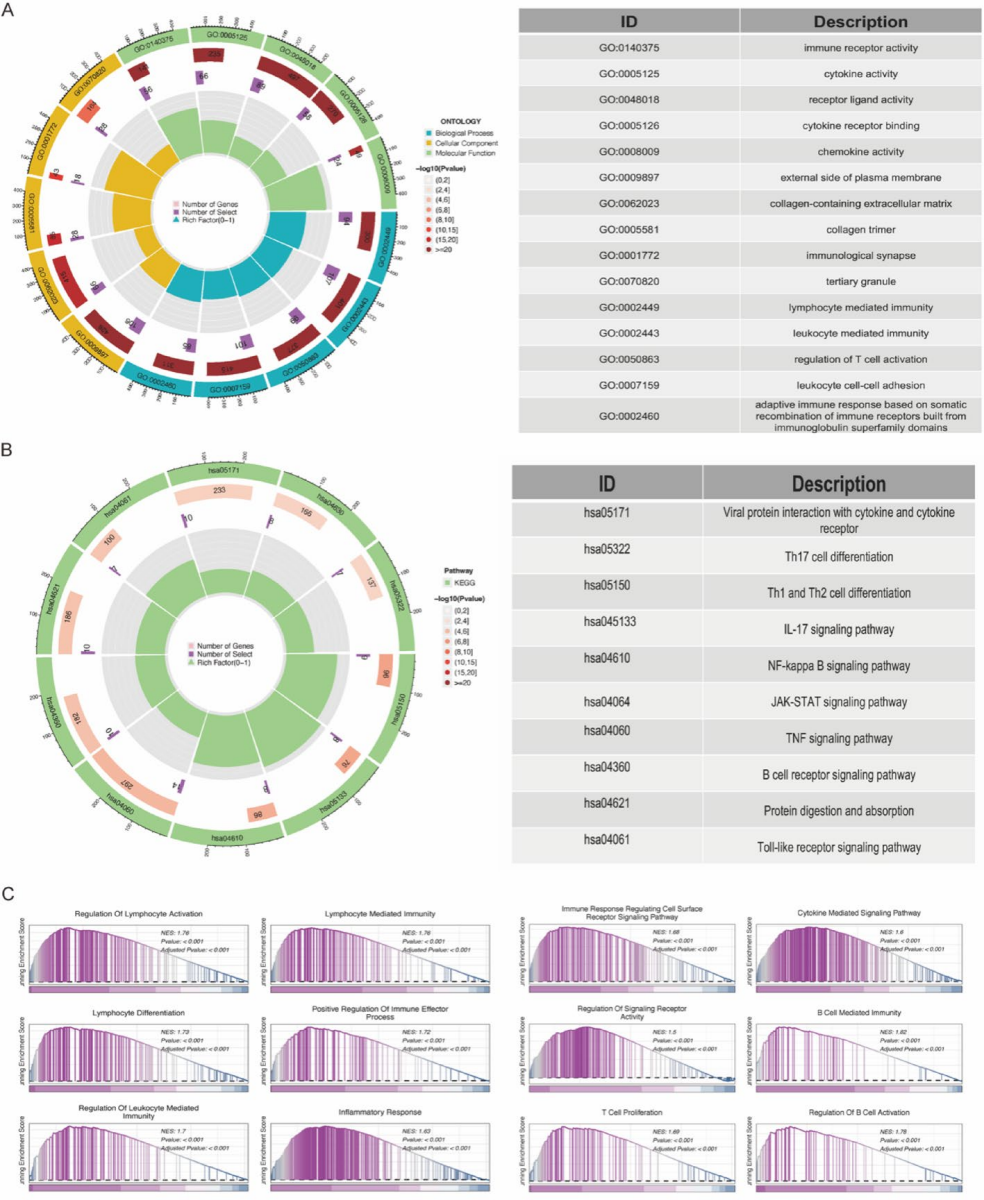
Enrichment Analysis.** A** GO enrichment analysis.** B** KEGG enrichment analysis.** C** GSEA enrichment analysis

Secondly, the enrichment of the IL-17 and Th17 cell differentiation pathways suggests that this gene may influence T cell polarization and play a critical role in the tumor immune microenvironment. Previous studies have demonstrated that the IL-17 signaling pathway promotes cancer cell proliferation and modulates the tumor-associated inflammatory environment across various tumor types.The results of this study further support that the target gene may mediate interactions between immune cells and the tumor microenvironment through this pathway (Fig. 9A and B).

Furthermore, GSEA results indicate that this gene is also significantly involved in B cell-mediated immunity and T cell proliferation, suggesting its potential impact on antigen presentation, adaptive immune responses, and immune memory formation (Fig. 9C). Notably, this gene exhibited widespread downregulation in pan-cancer analyses, a phenomenon that may be linked to the immune evasion mechanisms of cancer, highlighting its potential application in cancer immunotherapy.

### Immune infiltration analysis

**Fig. 10:**
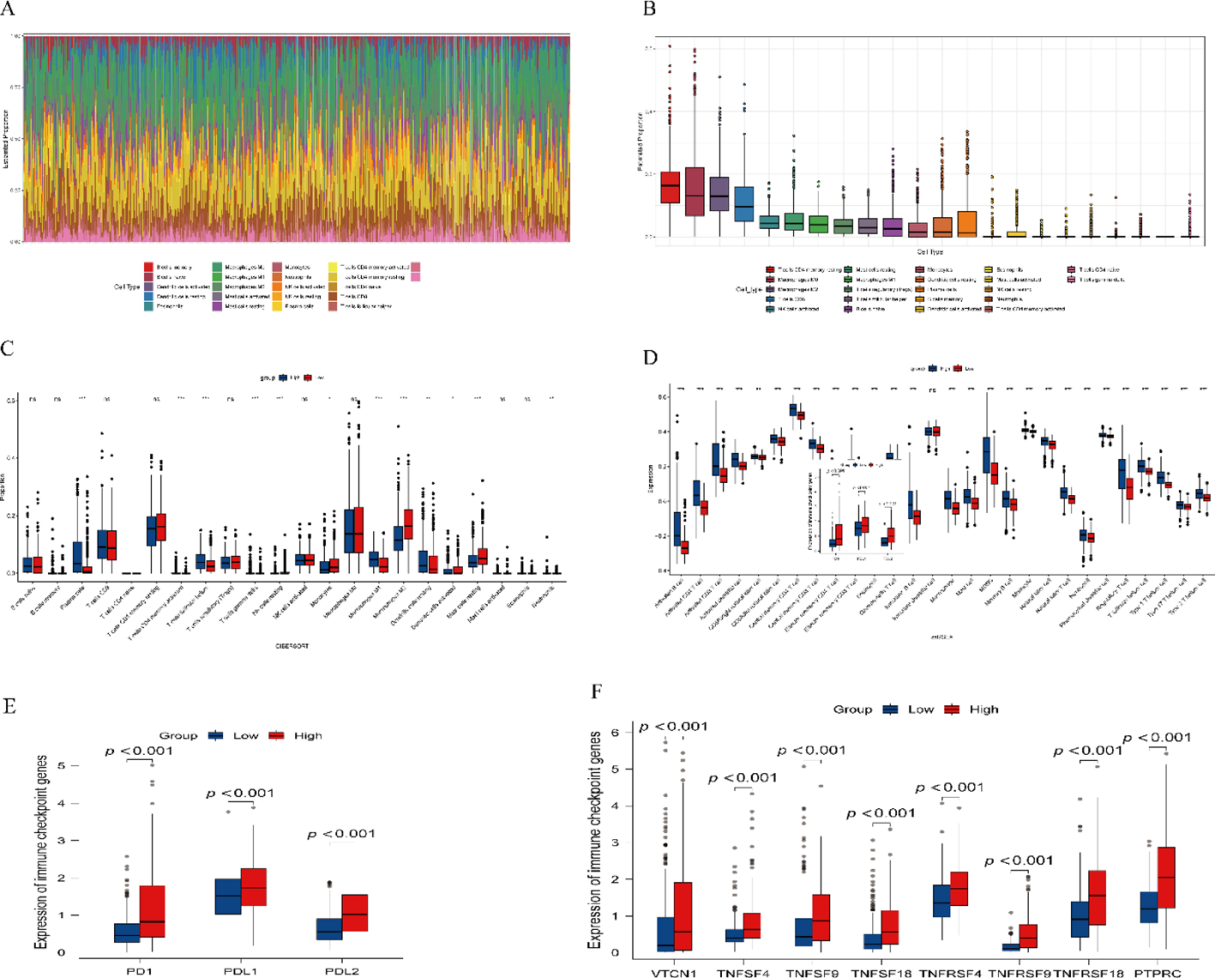
Immune Infiltration Analysis.** A**-**B** Immune cell abundance.** C** CIBERSORT analysis of immune cells.** D** ssGSEA immune cell analysis. **E**-**F** Immune checkpoint analysis

This study employed the CIBERSORT tool to analyze the abundance of 22 different tumor-infiltrating immune cell types (Fig. 10A and C). The results revealed that in the GPR132 low expression group, levels of monocytes, M2 macrophages, and resting mast cells were significantly higher (*P* < 0.05).Conversely, the GPR132 high expression group exhibited elevated levels of plasma cells, memory CD4 + T cells, follicular helper T cells, and M1 macrophages. High and low GPR132 expression groups were determined based on the median expression value across all samples, with samples above the median classified as high expression and those below the median as low expression. Using single-sample Gene Set Enrichment Analysis (ssGSEA), infiltration scores for 28 immune cell types were calculated, with detailed results presented in Fig. 10D. These findings underscore the pivotal role of tumor-infiltrating immune cells in the pathogenesis and progression of thyroid cancer (THCA).

Furthermore, differential expression analysis of immune checkpoint-related genes between the high and low GPR132 expression groups was conducted to investigate immune regulatory mechanisms (Fig. 10E and F). High GPR132 expression was associated with significantly increased levels of immune checkpoint-related genes, such as PD1 and CTLA4,suggesting that these patients may respond more effectively to immune checkpoint inhibitors.

### GPR132 suppresses tumor cell proliferation and invasion

**Fig. 11:**
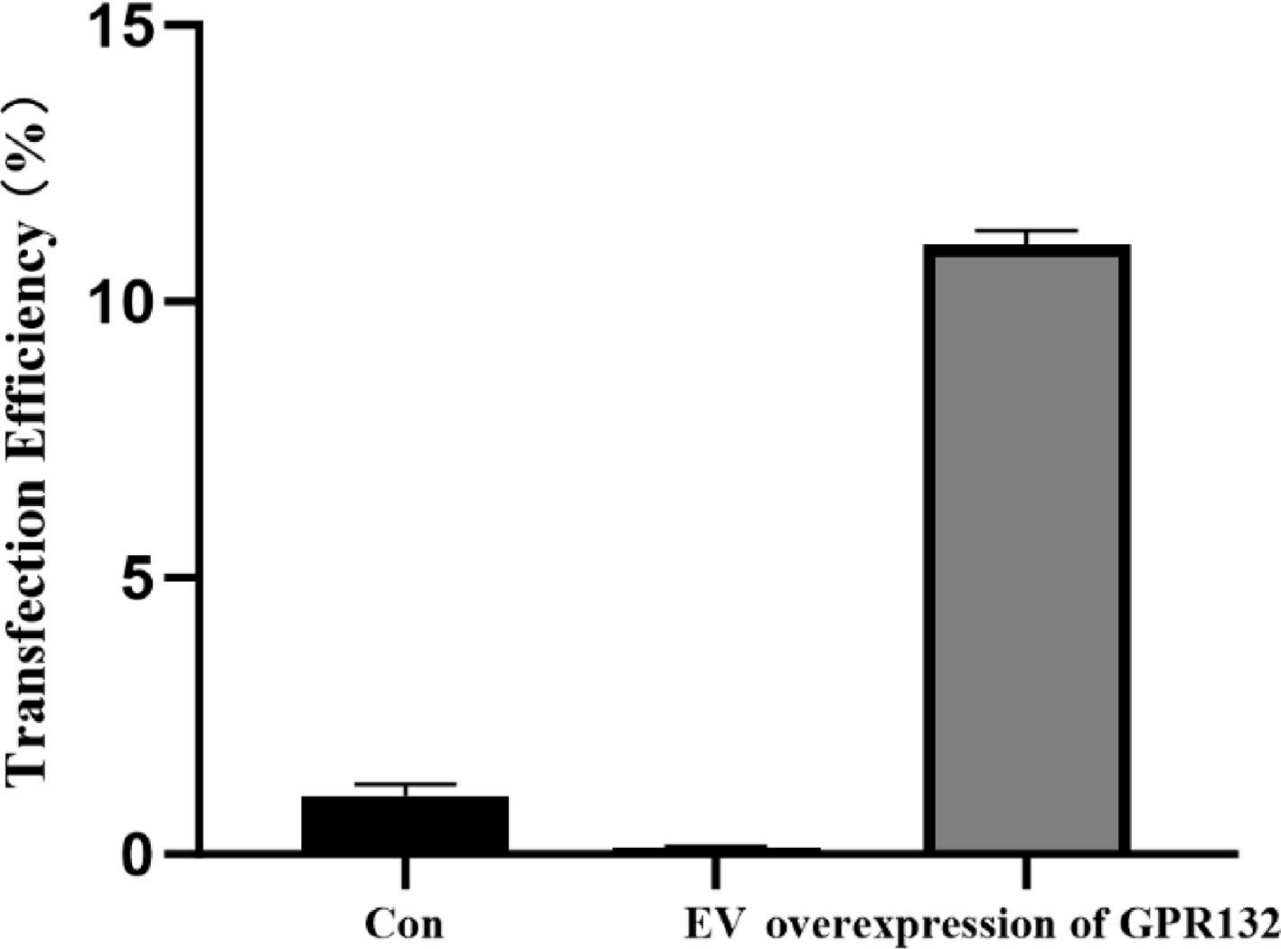
Transfection Efficiency of GPR132 in vitro

**Fig. 12:**
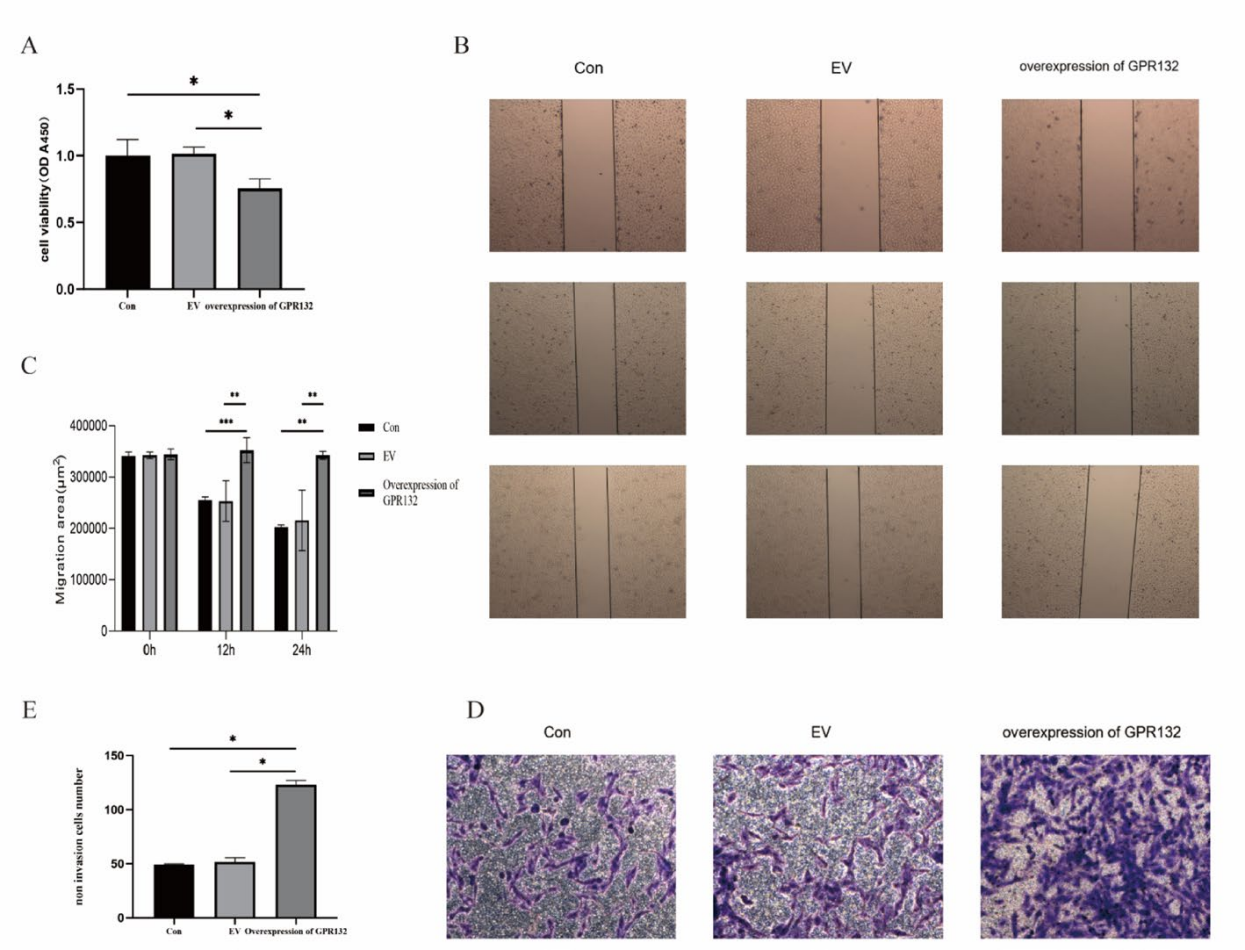
GPR132 Suppresses Tumor Cell Proliferation and Invasion.** A** Cell proliferation assay: overexpressing GPR132 exhibited significantly reduced proliferative capacity compared to the CON and EV (*p* < 0.05). **B** Wound healing assays CON: Initial scratch area measured (340,903 ± 4,641) µm², with final scratch areas of (254,989 ± 3,674) µm² at 12 h and (202,811 ± 2,272) µm² at 24 h, corresponding to a wound healing rate of 41% EV: Initial scratch area measured (342,388 ± 3,674) µm², with final scratch areas of (253,297 ± 8,372) µm² at 12 h and (215,746 ± 5,679) µm² at 24 h, corresponding to a wound healing rate of 40% GPR132 group: Initial scratch area measured (344,371 ± 4,080) µm², with final scratch areas of (352,496 ± 14,018) µm² at 12 h and (342,496 ± 4,337) µm² at 24 h** C** Wound healing assays: Compared to the CON and EV, overexpressing GPR132 exhibited a significant reduction in migratory capacity, with a 41% decrease in scratch wound healing rate (*p < 0.05)**D**-**E** Transwell migration and invasion assays

Building on the enrichment analysis results, we further investigated the functional role of GPR132 in thyroid cancer. First, we confirmed the transfection efficiency of GPR132 overexpression in TPC-1 cells, which was significantly higher than that of the control (CON) and empty vector (EV) groups (Fig. 11), indicating successful plasmid transfer. Subsequent functional assays revealed that GPR132 overexpression significantly suppressed tumor cell proliferation compared to the CON and EV groups (Fig. 12A). While wound healing assays showed no significant effect on cell migration (Fig. 12B and C), Transwell assays demonstrated that GPR132 overexpression significantly inhibited the migration capacity of TPC-1 cells (Fig. 12D and E). Collectively, these results suggest that GPR132 plays a suppressive role in tumor cell proliferation and invasion.

### GPR132 promotes cell apoptosis and affects cell cycle

**Fig. 13:**
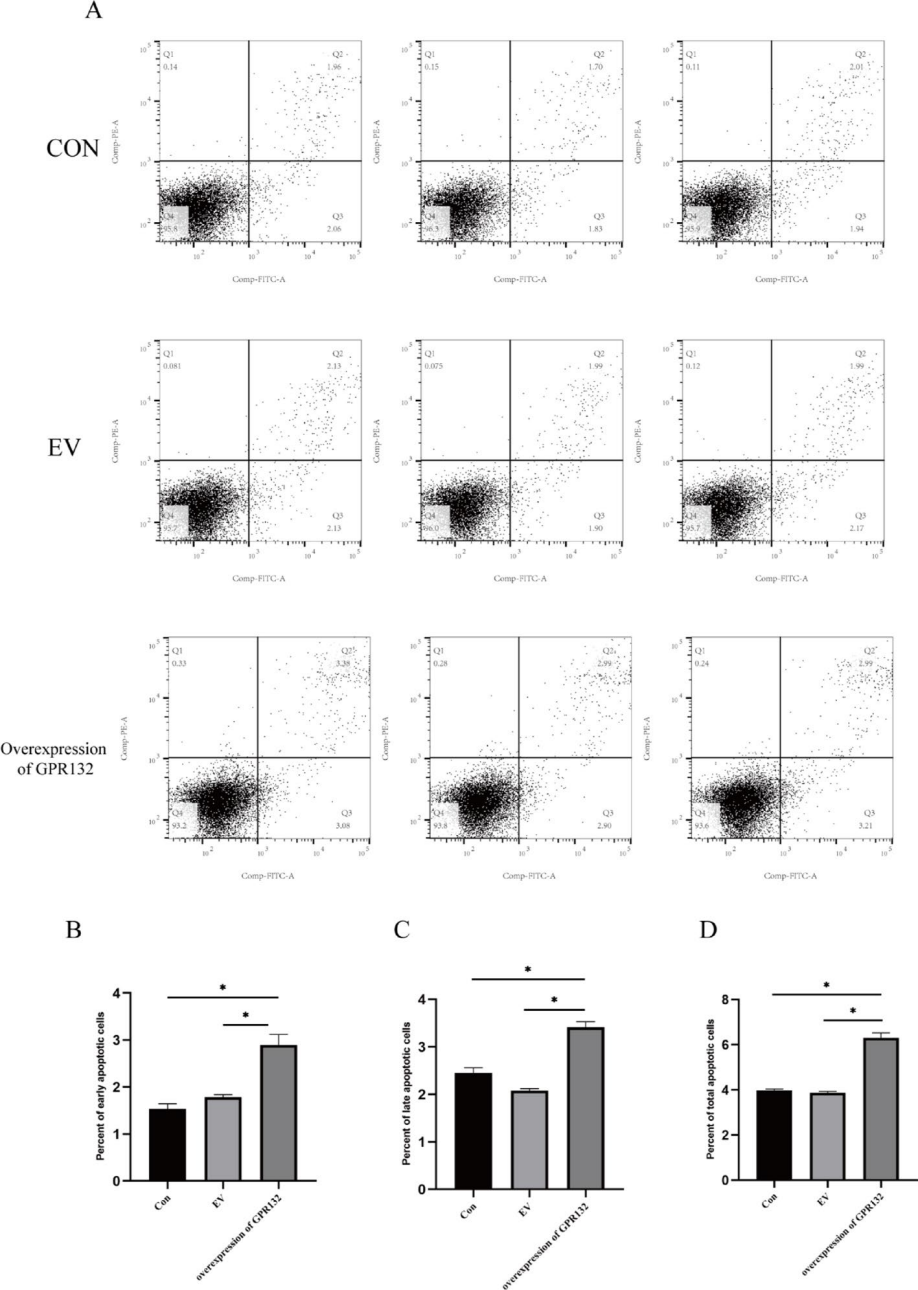
Flow Cytometry Analysis.** A** Apoptosis rates of THCA cells across groups.** B** Early apoptosis rate(The apoptosis rates in the CON, EV and overexpressing GPR132 were 1.54 ± 0.06%, 1.79 ± 0.03%, 2.90 ± 0.13%).** C** Late-stage apoptosis rate(The apoptosis rates in the CON, EV and overexpressing GPR132 were 2.44 ± 0.12%, 2.08 ± 0.04%, 3.41 ± 0.12%).** D** Total apoptosis rate(The apoptosis rates in the CON, EV and overexpressing GPR132 were 3.98 ± 0.05%, 3.87 ± 0.06%, 6.31 ± 0.21%)

**Fig. 14:**
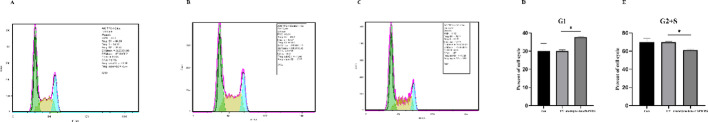
Cell Cycle Analysis. **A**-**C** Cell cycle distribution across groups.** D** G1 phase distribution comparisons(G1-phase percentages in the CON, EV and overexpressing GPR132 were 29.94 ± 4.32%, 29.98 ± 0.76%, 37.72 ± 0.22%). **E** G2 + S phase distribution comparisons

To further explore the impact of GPR132 on tumor cell apoptosis and cell cycle progression, we performed flow cytometry analysis.Our findings revealed that GPR132 overexpression markedly induced apoptosis in THCA cells (Figs. 13A–D).Additionally, GPR132 overexpression significantly impeded cell cycle progression by arresting cells in the G1 phase (Figs. 14A-E).

## Discussion

This study systematically characterized the cellular composition of the tumor microenvironment (TME) in papillary thyroid carcinoma (PTC) through single-cell RNA sequencing, revealing the pivotal role of tumor-associated macrophages (TAMs) in regulating tumor growth, metastasis, and immune evasion [20]. Based on GO and KEGG enrichment analyses, we identified that highly expressed genes in TAMs were significantly enriched in biological processes including T cell activation, immune response modulation, and MHC class I/II molecule-mediated antigen presentation. Consistent with prior findings, the elevated expression of MHC class I/II molecules (e.g., HLA-DR) substantially enhanced TAMs efficiency in capturing and presenting tumor antigens, thereby activating antitumor immune responses [21, 22].

We employed the “CellChat” R package to construct intercellular signaling networks and perform pseudotime analysis, revealing that the regulation of gene networks during pseudotime influences the polarization of tumor-associated macrophages (TAMs). The study demonstrates that ISG15 enhances antigen presentation by activating the STAT1 signaling pathway in the early stages of polarization, thereby promoting CD8⁺ T cell infiltration [23]. HES4, as an effector molecule of the Notch pathway, synergistically induces TNF-α/IL-6 release through TLR4 in the early phase, directly driving TAMs toward the M1 phenotype [24]. The TNFRSF4 gene dynamically modulates T cell co-stimulatory signals by suppressing Treg function and promoting Th1 responses, establishing an anti-tumor immune cycle. Its expression peak coincides with M1 markers, reflecting precise temporal regulation [23, 25]. These genes collaboratively drive TAMs toward the anti-tumor M1 phenotype, reshaping the immune microenvironment. Additionally, the TNF signaling pathway was found to play a pivotal regulatory role in macrophage differentiation and functional maturation. This finding aligns with previous research, indicating that the TNF signaling pathway has a dual role in the tumor microenvironment: on one hand, it enhances anti-tumor immune responses by activating immune cells such as M1 macrophages and memory CD4⁺ T cells; on the other hand, it supports tumor progression by promoting inflammatory responses and cell survival [26, 27].

Using weighted gene co-expression network analysis (WGCNA) and the CIBERSORTx algorithm, we identified genes associated with M1 and M2 macrophages and quantified their abundance in TCGA-THCA samples. Our analysis revealed that low levels of M1 macrophages are associated with improved prognosis in PTC patients. Although M1 macrophages are generally recognized for their anti-tumor effects, their excessive activation in certain tumor contexts may trigger heightened inflammatory responses, potentially promoting tumor progression or metastasis [28, 29].This observation underscores the context-dependent nature of macrophage polarization and function.

Furthermore, we intersected M1 macrophage module genes from TCGA-THCA with TAMs marker genes from the GEO database, identifying 82 M1-related TAM genes. Through a Venn diagram approach, we pinpointed GPR132 as a key prognostic gene. GPR132 exhibits diverse functions across tumor types: in breast cancer and melanoma, it promotes tumor growth and metastasis by modulating TAMs polarization [17, 30].in hematologic tumors, it exerts anti-tumor effects by regulating AML cell differentiation via the cAMP-PKA-mTOR signaling axis.This functional versatility suggests that the role of GPR132 may be contingent on the specific tumor type and its microenvironmental characteristics [8, 19, 30, 31].

In this study, low expression of GPR132 in PTC was significantly associated with poor prognosis. CIBERSORT analysis revealed that the GPR132 low-expression group exhibited heightened activity of immunosuppressive cell populations, including monocytes, M2-polarized macrophages, and resting mast cells, collectively promoting tumor immune evasion [32, 33]. In contrast, the GPR132 high-expression group demonstrated significantly increased infiltration of anti-tumor effector cells, such as M1 macrophages, memory CD4 + T cells, and follicular helper T cells, a cellular profile closely linked to favorable clinical outcomes [34].The distinct tumor-suppressive role of GPR132 in papillary thyroid carcinoma (PTC), as opposed to its oncogenic functions observed in breast cancer and melanoma, fundamentally stems from PTC unique tissue-specific signaling context. As a malignancy originating from thyroid follicular epithelium, Within this context, GPR132 likely activates the TNF-α-mediated caspase-8/caspase-3 apoptotic pathway through direct interaction with key molecules in the TNF signaling cascade, while simultaneously enhancing anti-tumor immunity by promoting M1 macrophage polarization in the tumor microenvironment. This signaling interaction pattern exhibits high compatibility with the TNF pathway-dominated apoptotic regulation characteristic of PTC, ultimately rendering GPR132 a tumor suppressor rather than an oncogene in PTC. Pathway enrichment analysis further corroborates this mechanism, demonstrating GPR132 significant regulatory effect on the TNF signaling pathway and suggesting its potential influence on TNF-mediated apoptosis through expression-level modulation.

To integrate theoretical predictions with empirical evidence, we conducted in vitro validation experiments to corroborate the computational results. This study systematically evaluated the impact of GPR132 overexpression on the biological behavior of TPC-1 cells by constructing a GPR132 overexpression system. In the cell proliferation assay, the absorbance at 450 nm in the overexpression group significantly decreased from a baseline of 1.0 to 0.76 after 24 h of culture (*p* < 0.05), indicating that GPR132 overexpression inhibits tumor cell proliferation(Fig. 12A). The scratch assay further revealed that the scratch area in the overexpression group remained unchanged at 340,000 μm² over 24 h, whereas the control group’s area decreased to 200,100 μm² (*p* < 0.05), confirming that GPR132 overexpression reduces cell migration rate by 41%(Fig. 12B-C). Transwell assay results showed that the number of non-invasive cells in the GPR132 overexpression group was higher than in the CON and EV groups, consistent with the findings that high expression of the GPR132 gene simultaneously inhibits cell migration and invasion capabilities((Fig. 12D-E). These findings suggest that GPR132 may exert its anti-cancer effects by regulating cell motility-related signaling pathways. Additionally, flow cytometry analysis demonstrated that GPR132 overexpression promotes tumor cell death by inducing G1 phase cell cycle arrest and activating the extrinsic apoptosis pathway (Fig. 13A-D). Although no significant difference was observed between the CON and overexpression groups due to increased intra-group variability, G1 phase arrest was evident. These findings indicate that GPR132 may play an anti-tumor role by modulating the balance between cell cycle and apoptosis, although the specific mechanisms require further investigation.

The study reveals that GPR132 overexpression induces G1 phase arrest in TPC-1 cells, a phenomenon potentially mediated by the synergistic interplay between cell cycle regulatory molecules and the immunomodulatory functions of tumor-associated macrophages (TAMs), ultimately influencing tumor progression. Specifically, GPR132 upregulation elevates p21 expression while suppressing the activity of cyclins (e.g., Cyclin D), a regulatory pattern that aligns with GO/KEGG enrichment results of highly expressed genes in TAMs subsets (e.g., immune activation and antigen presentation pathways). Mechanistically, this interaction network likely operates through bidirectional signaling: On the one, GPR132-overexpressing tumor cells secrete specific cytokines during proliferation, significantly upregulating MHC-II molecule expression in TAMs. As critical mediators of tumor antigen presentation, MHC-II molecules effectively promote CD4^+^T cell activation, thereby enhancing the regulatory hub function of antitumor immune responses; Other handActivated TAMs reciprocally upregulate p21/p27 expression in tumor cells via cytokine and metabolite secretion, further inhibiting Cyclin D and downstream CDK4 activity. This dual mechanism reinforces G1 phase arrest in tumor cells, establishing a cell cycle regulation-immune activation positive feedback loop that collectively suppresses tumor progression.

In cancer, the growth and death patterns of tumor cells, along with the polarization of TAMs in the tumor microenvironment, are critical for predicting tumor recurrence and invasiveness. G protein-coupled receptors (GPCRs) play a pivotal role in tumor initiation, promotion, and metastasis by activating major proliferation and survival signaling pathways associated with cancer cells [35–37]. This study highlights the potential prognostic value of GPR132 in PTC and provides preliminary insights into its mechanisms within the tumor microenvironment. However, as this study primarily focused on screening prognostic genes using single-cell sequencing, the functional research on GPR132 remains in its early stages. Future studies will further elucidate the specific signaling pathways regulated by GPR132 and its potential applications in precision therapy for PTC.

In conclusion, this study uncovered the potential prognostic value of GPR132 in PTC through single-cell sequencing and functional experiments, offering preliminary insights into its mechanisms within the tumor microenvironment. These findings provide novel perspectives and methodologies for the precise diagnosis and individualized treatment of PTC, while also serving as an important reference for research on the functions of GPR132 in other tumor types.

## Conclusion

In summary, GPR132 has been identified as a key prognostic gene for PTC. In vitro experiments have shown that overexpression of GPR132 can reduce the proliferation and migration abilities of TPC-1 cells, and may induce apoptosis through the extrinsic apoptotic pathway. However, the normal tissue samples used in this study were obtained from one side of the thyroid of a single patient, and the single cell line might introduce limitations. In subsequent studies, we will include more normal tissue samples and cell lines with different driver mutations for verification to increase the universality of the results Table 1.

**Table 1:** Primer sequences

Gene	Primer (5′-3′)
β-actin	F: GGCTGTATTCCCCTCCATCG
	R: CCAGTTGGTAACAATGCCATGT
GPR132	F: GGTGGTTITCATCTTCCTAGTC
	R: CGTTCCTGTCTCCTCTGTAGTA

Note: F/R: Forward primer / Reverse primer;

β-actin: Reference gene used for normalization of qPCR data;

The primers were designed based on NCBI GeneBank

## Data Availability

The data analyzed in this study are available from the [NCBI]database.The direct links required to find each data set in the database are as follows: theGEO gene expression and clinical pathology data set: https://www.ncbi.nlm.nih.gov/geo/query/acc.cgi? acc=GSE191288.Thedata set downloaded by this direct link is the original data set.

## Abbreviations

PTC: Papillary thyroid carcinoma
TAMs: tumor-associated macrophages
TPC-1 cell: Thyroid Papillary Carcinoma 1 cell
GPCR: G protein-coupled receptor
AML: acute myeloid leukemia
PPAR-γ: peroxisome proliferator-activated receptor gamma
ALL: acute lymphoblastic leukemia
